# Supplementary material for: Medical students’ knowledge of and attitudes towards LGBT people and their health care needs: Impact of a lecture on LGBT health
Source: PLoS One. 2020 Jul 1;15(7):e0234743. doi: 10.1371/journal.pone.0234743 (PMC7329058; doi:10.1371/journal.pone.0234743)
Supplement: S1 Appendix — (DOCX) [file pone.0234743.s001.docx]

**Appendix 1: Outline of one-hour lecture on sexual orientation and gender identity development**

1. Recognize the place of sexuality in the developmental process during adolescence

- Developmental perspective on sexuality
- Questions regarding sexual orientation during consultation
- Dimensions of gender identity

1. Describe the situation and trends in sexual orientation and behavior

- Prevalence of non-heterosexual orientation and transgender identity
- Health and social challenges facing these groups—e.g., increased risk of suicidality

1. Addressing sexuality with teens during consultation

- Ask about knowledge, attitudes, sexual orientation, satisfaction/pleasure, contraception, abuse
- Evaluate facets of gender identity
- Common obstacles on the part of teens
- Common obstacles on the part of practitioners
- Social support by family and peers

1. Some current issues

- Sexual dysfunction
- Transgender
  - hormone blockers
  - reassignment surgery
  - medical follow-up
- Homosexuality
  - HIV & STI
  - functional disorder symptoms
  - depression & anxiety
- Support for parents

1. Take-home messages
2. Useful references

**Appendix 2: Items measuring knowledge of, attitudes towards, and experiences with LGBT people as used in the actual survey in French**

1. Les adolescent-e-s LGBT ont les mêmes besoins en matière de santé que les adolescent-e-s qui ne sont pas LGBT.

2. La différence entre orientation sexuelle et identité de genre est claire pour moi.

3. Les homosexuel-le-s sont habituellement reconnaissables par leur apparence et leurs manières.

4. Je fréquente régulièrement des personnes LGBT dans ma vie de tous les jours.

5. Les couples homosexuels devraient être autorisés à adopter des enfants.

6. La chirurgie de réassignation sexuelle est facilement disponible pour les personnes trans et couverte par l’assurance maladie.

7. Je pense que l’homosexualité est immorale.

8. Les associations qui défendent les droits des personnes LGBT sont nécessaires.

9. En tant que médecin, je pense qu’il est important d’inclure dans l’anamnèse de mes patients des questions sur leur vie affective et sexuelle, leur orientation sexuelle et leur identité de genre.

10. Si j’ai le choix, je préfère ne pas prendre en charge une personne gay, lesbienne ou bisexuelle.

11. Lorsque je rencontre un-e collègue ou un-e patient-e, je pars en général du principe qu’il/elle est hétérosexuel-le.

12. Mes rencontres et mes discussions avec des personnes LGBT ont influencé positivement mes perceptions concernant les personnes LGBT.

13. Les patientes lesbiennes n’ont pas besoin d’un frottis du col utérin aussi souvent que les femmes hétérosexuelles.

14. Si j’ai le choix, je préfère ne pas prendre en charge une personne transgenre.

15. Les couples homosexuels devraient être autorisés à se marier.

16. Changer le sexe d’un individu (hormone et/ou chirurgie) est contre mes valeurs morales.

17. S’identifier comme transgenre doit être considéré comme une pathologie psychiatrique.

18. Dans notre société, les jeunes LGBT sont actuellement bien acceptés et n’ont donc pas de barrières à l’accès aux soins.

19. Imaginer des personnes de même sexe avoir des relations intimes me met mal à l’aise.

20. Je me sentirais mal à l’aise d’examiner et de soigner une personne de mon sexe qui est homosexuelle.

21. Les hommes homosexuels sont généralement efféminés et les femmes homosexuelles généralement masculines.

22. Etre homosexuel est un choix.

23. Les tentatives de suicide concernent les adolescents LGBT dans des proportions identiques à celles observées chez les adolescents hétérosexuel-le-s.

24. Je serais à l’aise avec le fait que mes collègues apprennent que je m’occupe de patient-e-s LGBT.

25. Le cancer du sein peut encore survenir après une chirurgie bilatérale de réduction mammaire pour les hommes transgenres.

26. Les programmes d’éducation sexuelle à l’école devraient aborder toutes les orientations sexuelles.

27. Les adolescent-e-s LGBT ont plus tendance à consommer de l’alcool, du tabac ou d’autres substances psychoactives que les autres adolescent-e-s.

28. Les gays et les lesbiennes ont une prévalence plus élevée d’anxiété et de dépression par rapport aux personnes hétérosexuelles.
